# Effects on different full-coverage designs and materials of crack propagation in first mandibular molar: an extended finite element method study

**DOI:** 10.3389/fbioe.2023.1222060

**Published:** 2023-08-15

**Authors:** Jianzhao Ni, Liang Xu, Yunzhi Lin, Danlin Lai, Xiaohong Huang

**Affiliations:** Department of Stomatology, The First Affiliated Hospital, Fujian Medical University, Fuzhou, China

**Keywords:** extended finite element method, cracked tooth, biomechanics, crack propagation, restoration design

## Abstract

**Objectives:** This study aims to investigate the biomechanical properties of fracture resistance in cracked teeth using five different full-coverage restorations made of three different materials.

**Materials and Methods:** A 3D model of a mandibular first molar was created to design five different full-coverage repair models: crown, crown with composite resin filling inside, occlusal veneer, occlusal veneer with composite resin filling inside and onlay. These repair models were fabricated using three different materials, namely, zirconia, lithium disilicate (LDS), and a hybrid polymer-infiltrated ceramic network material (PIC). In total, 15 repair models were tested using the extended finite element method (XFEM), with an occlusal load of 5000 N applied slowly to the occlusal surface of the restoration. The analysis of stress distribution in the restoration and dentin crack line was conducted to measure and record the crack initial load on the restoration and dentin.

**Results:** The results showed that restorations on the occlusal surface significantly improved crack resistance, with zirconia exhibiting superior fracture resistance among the materials tested. Restorations of crown with composite resin filling inside demonstrated the highest resistance to fracture, while occlusal veneers showed the lowest. MPS concentration was observed at the interface between the restoration and dentin and at the root bifurcation, with the highest values at the top of crack development. Dentin covered by oxidized restorations had the highest displacement, while PIC restorations exhibited the lowest. Pulp analysis revealed selective MPS concentration and strain patterns in models with zirconia restorations and onlay, with pronounced pulp displacement in zirconia restorations and onlay. Enamel analysis indicated larger MPS values and displacements in zirconia restoration models and onlay, with higher strain in onlay. Restoration played a crucial role in protecting the tooth, with crack propagation initial loads in dentin surpassing restorations in experimental groups.

**Conclusion:** This study confirms that full-coverage restorations significantly increased the fracture resistance of cracked teeth, with zirconia restorations significantly protecting the underlying cracked tooth. Elimination of fracture lines in the restoration design can improve fracture resistance in cracked teeth. The findings have implications for dental prosthetic design and clinical practice.

## Introduction

A cracked tooth is a common dental condition, with as many as 70% of patients who have received dental treatment exhibiting at least one posterior tooth with a significant crack (Hilton et al., 2011). This condition is characterized by an incomplete fracture that originates at the occlusal surface and extends mesiodistally and subgingivally (Leong et al., 2020). It is more prevalent among adults, particularly in mandibular molars (Krell and Caplan, 2018). If left untreated, the fracture may invade the pulp chamber, leading to an infected dental pulp (Türp and Gobetti, 1996; Ricucci et al., 2015). Therefore, early diagnosis and treatment are essential to prevent progression and preserve dental pulp vitality (Aquilino and Caplan, 2002; Wu et al., 2019; de Toubes et al., 2022).

While various materials and designs have been employed to treat cracked teeth, there is no universally accepted gold standard for treatment (Guthrie and DiFiore, 1991; Davis and Overton, 2000; Opdam and Roeters, 2003; Banerji et al., 2010). Previous investigations have revealed significant variations in treatment planning approaches among practitioners and specialists (Abulhamael et al., 2019; Hilton et al., 2020). The selection of an appropriate clinical modality and suitable materials is crucial for achieving favorable outcomes in the management of cracked teeth. Direct composite resin restorations, particularly with cusp coverage, have shown high success rates in the treatment of painful cracked teeth, as evidenced by a 7-year clinical study (Opdam et al., 2008). Notably, no failures were observed in teeth with cusp coverage. Similarly, indirect resin composite restorations and early full-coverage crowns have demonstrated favorable survival rates in other investigations. For instance, a 6-year survival rate of 93.02% was reported for indirect resin composite restorations of painful cracked teeth (Signore et al., 2007). Full-coverage restorations are particularly effective in suppressing crack expansion and reducing the incidence of periodontal disease associated with occluded fissures (Krell and Rivera, 2007; Abulhamael et al., 2019). A retrospective clinical study shows early full-coverage crowns provide protection to cracked teeth and improve survival rates (Kanamaru et al., 2017). Another study reported a survival rate of 89% over a 60-month follow-up period using gold occlusal veneers to treat cracked teeth (Chana et al., 2000). Although these studies have shown promising clinical efficacy, there is a lack of cross-sectional comparisons among studies and different materials and designs, as well as a limited understanding of their internal biomechanics.

The debate surrounding crack elimination *versus* retaining crack lines adds complexity to the management of cracked teeth. Some researchers advocate for crack elimination using resin cement with an elastic modulus similar to dentin to alter stress distribution and prevent stress concentration in the affected area (Abbott and Leow, 2009; Mahgoli et al., 2019). On the other hand, proponents of retaining crack lines argue that full-coverage restorations can provide clamping support for cracked teeth and effectively halt further crack development (Soares de Toubes et al., 2020). As a result, the optimal approach for managing cracked teeth remains an area of ongoing research and clinical exploration. Establishing a standardized approach backed by comprehensive research and biomechanical understanding is crucial for providing optimal care to patients with cracked teeth.

Recent advancements in CAD-CAM dental materials, such as resin composites, glass ceramics, and zirconia, have significantly improved restorative and aesthetic dentistry. These materials offer superior precision fit, enhanced mechanical performance, and the ability to faithfully replicate the natural appearance of teeth (Ausiello et al., 2017; May et al., 2022). Zirconia, known for its exceptional fracture resistance and mechanical strength, is a popular choice for reinforcing weak posterior teeth exposed to high biting forces. Its use in cracked tooth restoration effectively reduces crack propagation and safeguards against fractures during chewing. However, challenges may arise in stress distribution when zirconia is bonded to natural teeth due to its high modulus of elasticity. Nonetheless, its durability provides reliable protection for tooth tissues (Pjetursson et al., 2018; Tang et al., 2019). Lithium disilicate (LDS) is favored for restoring anterior teeth with moderate forces due to its favorable aesthetics and moderate fracture resistance. In cases with higher occlusal forces, zirconia may be preferred for its excellent fracture resistance. LDS, with its lower modulus of elasticity closely resembling that of natural teeth, promotes better stress distribution and minimizes the risk of damage to surrounding tissues (Sulaiman et al., 2020). Hybrid polymer-infiltrated ceramic network material (PIC), combining ceramics and polymers, offers enhanced toughness and resilience compared to traditional ceramics. It effectively resists fractures, withstands occlusal forces, and protects dental tissues, with a modulus of elasticity similar to that of natural teeth, proficiently absorbing and dispersing stress and safeguarding dental structures (Facenda et al., 2018; Machry et al., 2021). Ultimately, the choice of material—zirconia, LDS, or hybrid PIC—depends on specific restoration requirements, as there is no established gold standard. The continuous advancements in CAD-CAM dental materials are shaping modern dentistry, empowering dentists to achieve optimal outcomes in restorative and aesthetic dental treatments.

Finite element analysis is a powerful tool for studying dental biomechanical processes, particularly in simulating and predicting the response of materials to different forces (Dal Piva et al., 2018; Tribst et al., 2018; Waldecker et al., 2019). However, traditional finite element analyses often used in dental biomechanical studies are limited in modeling dental mechanical processes comprehensively by quantifying the stress-strain distribution in the structure. These studies lack validity in terms of fracture mechanisms and failure principles that lead to the rupture of dental structures (Li et al., 2004). The extended finite element method (XFEM) can analyze crack behavior and solve for parameters such as stress intensity factors at the crack tip, independently of the finite element mesh (Wan et al., 2019). Previous studies have already been conducted to apply extended finite elements to dentistry research. Innovators have employed the XFEM to demonstrate that cracks with significant bending configurations or larger crack tip angles enhance the fracture resistance of dental enamel (Shahmoradi et al., 2020). Additionally, researchers have utilized XFEM to investigate the impact of different pulpal cavity access openings on the fracture resistance of teeth (Zhang et al., 2019). The study concluded that XFEM can be applied to the study of cracked teeth to obtain more comprehensive results, as it not only simulates the stresses in the material but also predicts the trend of fracture expansion.

This study aims to comprehensively investigate the expansion trend of cracks in mandibular first molars after restoration using different approaches and materials. Specifically, five different full coverage methods will be utilized, with three of them involving the elimination of partial crack lines, while the other two retain the crack lines. The materials used in the study will include zirconia, LDS, and PIC. To conduct this investigation, the XFEM will be employed within the Abaqus software, enabling a thorough biomechanical analysis of the cracked teeth. By examining the impact of various restorative techniques and materials on cracked teeth, this study aims to provide valuable insights and a theoretical basis for future long-term follow-up trials in cracked tooth management. The results obtained from this research will offer potential options and guidelines for effectively treating cracked teeth and preserving their long-term integrity.

## Material and methods

### Creation of the geometric model

In this study, a cavity-free mandibular first molar was randomly selected as the prototype of the study model. After obtaining approval from the Ethics Committee of the First Hospital of Fujian Medical University, the tooth was scanned using Cone beam CT (Cranex 3D, 3D Dental Imaging System, Kava, USA). The scanning parameters were as follows: 70 kV, 100 mA, 0.3 mm slice thickness, and the data were converted to Digital Imaging and Communications in Medicine (DICOM) file.

The DICOM format file was imported into MIMICS 21.0, and the tooth area was filtered according to the grayscale value. Then, the tooth was segmented using the mask tool to obtain a preliminary 3D model that included enamel, dentin, pulp, periodontal membrane, and alveolar bone. The resulting preliminary 3D model of the tooth was exported to an STL format and imported into Geomagic 13.0 software for further optimization. In Geomagic 13.0, the model’s holes were filled, and the sharp corners were smoothed, based on the original curvature of the selected area. This polishing did not affect the model’s significant features. The smoothed model was then divided into surface slices. Finally, the 3D solid model was synthesized and exported into an IGES format file. The periodontium was formed by offsetting the teeth outward by 0.2 mm in Geomagic 13.0. The crown and dentin were also divided.

Next, the model was saved as a Standard for the Exchange of Product Model Data (STEP) file and imported into Hypermesh (v 2019, Troy, MI), where the cells were divided into crowns, dentin, periodontal membrane, pulp, alveolar bone, and restorations. The cell type used was tetrahedral C3D4 cells, with a total of 261,700 meshes and 57,300 nodes, and an average edge length of 0.3 mm (Table 1).

**TABLE 1:** Restoration design and material of experimental six groups.

Group	Restoration design	Whether to eliminate crack lines	Total # of nodes	Total # of element
Group 1 (Control group)	—	No	84106	412188
Group 2	Crown	No	57312	261762
Group 3	Crown, composite resin filling inside	Yes	58826	267432
Group 4	Occlusal veneer	No	66892	317783
Group 5	Occlusal veneer, composite resin filling inside	Yes	67976	320359
Group 6	Onlay	Yes	67976	320359

**FIGURE 1:**
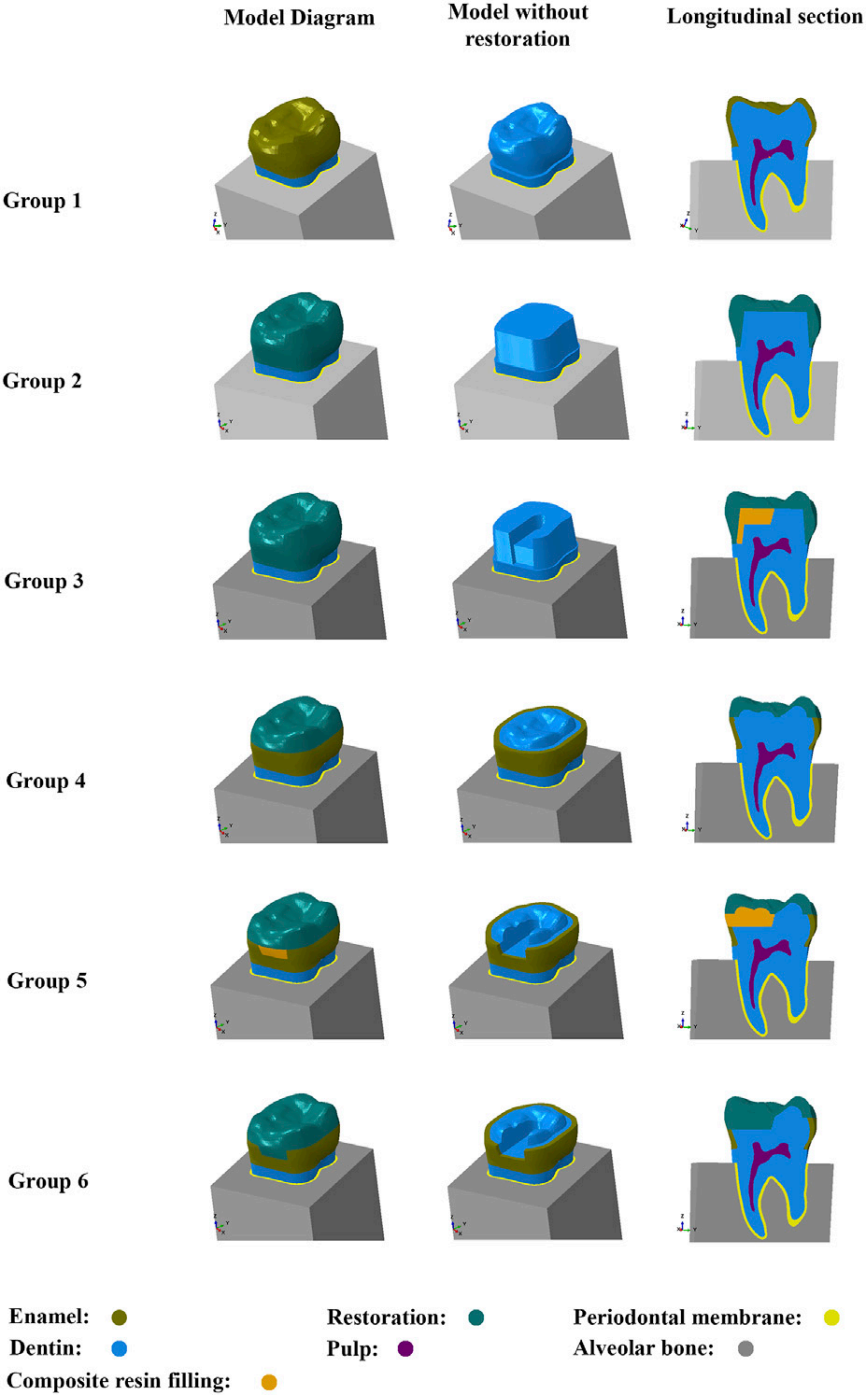
The control group and the composition of the five restoration models:**Group 1**: control group; **Group 2**: crown; **Group 3**: crown and composite resin filling inside; **Group 4**: occlusal veneer; **Group 5**: occlusal veneer, composite resin filling inside; **Group 6**: onlay.

In total, 15 restorations were applied to the same cracked tooth model. Five restorative designs (full crown, Occlusal veneer, and onlay) (Table 1)and three combinations of restorative materials [zirconia, lithium disilicate (LDS), and hybrid polymer-infiltrated ceramic network material (PIC)] were used. The crown’s design included an 8° taper of the axial wall and chamfered margins that ended at the pulp-enamel junction. There were two types of crowns: those with and without direct composite resin filling. After removing the fissure line, the direct composite resin filling was applied inside the crown. The cavity type inside the crown was entered in (Figure 1). Two types of Occlusal veneers were used, including crowns with or without direct composite resin filling. A slight modification was made to the composite resin filling inside the occlusal veneer, and its internal structural data are shown in (Figure 1). For the veneer design, the internal resin filling of the occlusal veneer was integrated with the restoration on the occlusal surface, and the occlusal veneer model was then formed. In total, 15 models were generated by combining three different restorative models with or without the internal composite resin filling.

### Loading conditions

**FIGURE 2:**
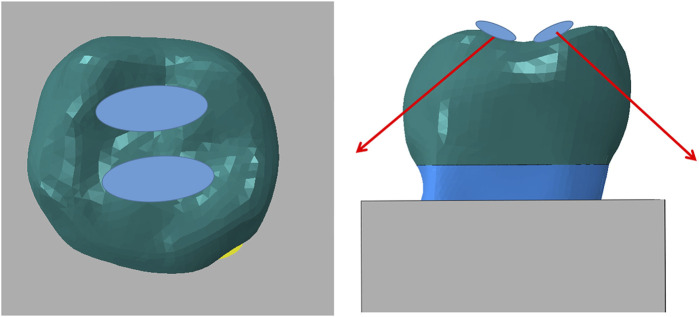
Loading conditions.

Based on previous studies, this experiment utilized three-dimensional extended finite elements to analyze complex structures with multiple parts. The loading conditions were applied to the occlusal surface using the principle of engineering equivalence, with a diagonal downward load of 45° on both sides. The analysis was conducted using crack propagation analysis in early ABAQUS software (v 2021; SIMULIA, France), with a load of 5,000 N applied in two fractions to two surfaces of the tooth tip, simulating the force applied when biting a hard object (Figure 2).

### Making original cracks

**FIGURE 3:**
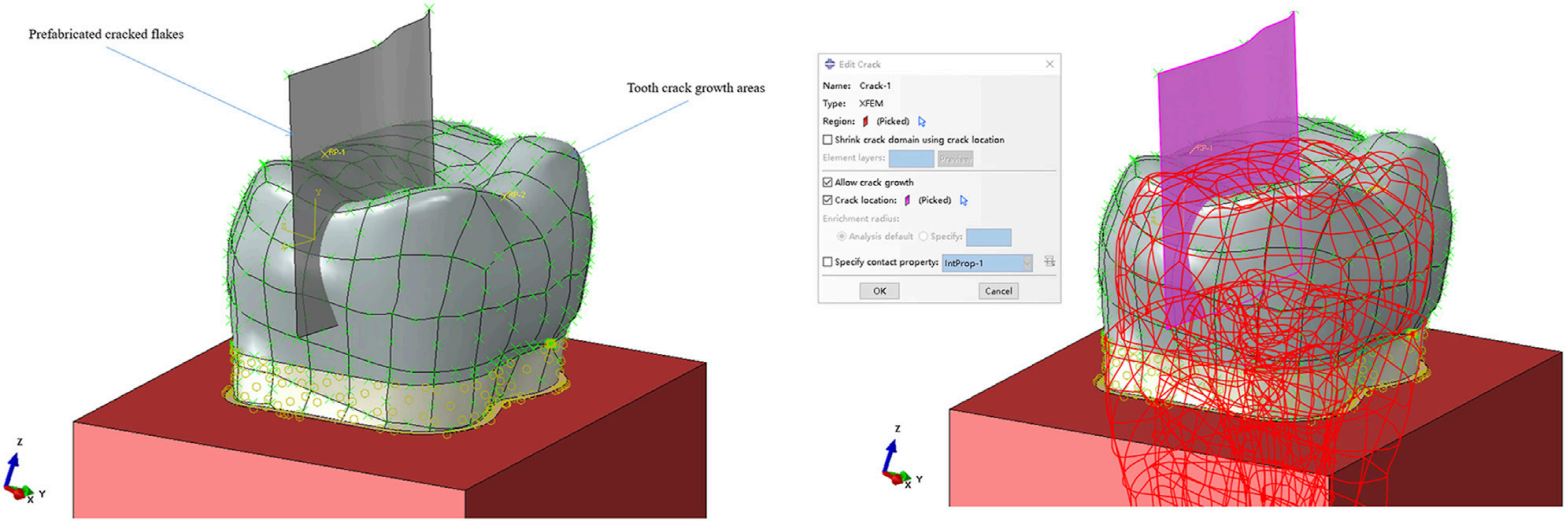
Initial test crack setting.

The cracks used in the study were prefabricated cracks, which were created using a thin slice in Abaqus to replace the initial form of the crack. This slice was positioned in contact with the area of the crack, resulting in the generation of the initial crack. The cracks extended through the axial surface of the tooth and partially into the buccal surface, reaching the dentin layer at a depth of 2 mm from the pulp chamber. It is important to note that the cracks did not involve the dental pulp. This method was employed to generate the initial crack for both the control group for the crown and dentin, as well as for the dentin in the restoration protocol (Figure 3).

## XFEM

The properties of the materials are shown in Table 2. In this analysis, we have made a number of assumptions to simplify the modeling process and provide insights into the behavior of the system. Specifically, we have assumed that the material being analyzed is homogeneous, isotropic, and elastic and that all tie-rod connections are used for additional support.

**TABLE 2:** MOE and Poisson’s ratio of enamel, dentin, and various restoration materials.

	Young’s modulus (GPa)	Poisson’s ratio	Tensile stress δTS (Mpa)	Fracture toughness KIC (Mpa·m1/2)	Strain energy release rate G (J/m2)
Enamel	83.5	0.27	11.5	3.1	115.1
Dentin	18.6	0.31	41.3	2.2	260
Pulp	2	0.45	—	—	—
Zirconia	206.5	0.259	865	5	121.1
Lithium disilicate (LDS)	103	0.215	210	2.27	50
Hybrid polymer-infiltrated ceramic network material (PIC)	38.08	0.233	148	1.2	37.8
Filling composite resin (Filtek Z350)	9.8	0.25	—	—	—

We have also assumed that the dentin initially contains cracks and that these cracks can grow on both the crown and the dentin. We have not included an adhesive resin layer in our analysis, as we have assumed that the interface between each component is fully bonded. Additionally, we have assumed that all dentin is anchored to the base of the alveolar bone.

To relate the critical strain energy release rate G (J/m^2^) to the fracture toughness KIC (MPam1/2) a key material parameter that represents the strain energy required per unit area for crack expansion, we have used the following equation within the domain of online elastic fracture mechanics:

$$G=\frac{K_{IC}^{2}}{E}$$

where E is the Young’s modulus and *μ* is Poisson’s ratio. By making these assumptions and using this equation, we are able to gain valuable insights into the fracture behavior of the system being analyzed. The crack initial load of restoration and dentin in each case was measured.

In our study, we employed the XFEM module of Abaqus to conduct a comprehensive analysis of crack extension. The underlying formulas and principles governing the XFEM cohesive segments in our model bear a remarkable resemblance to those governing cohesive units exhibiting traction-separation intrinsic behavior. Furthermore, this analogy can be extended to encompass the linear elastic traction-separation model, as well as the criteria for damage initiation and its subsequent evolution. Specifically, we adopted the Maximum Principal Stress (MPS) criterion as the basis for determining the initiation of damage in the material under investigation. This criterion can be mathematically expressed as follows:

$$f^{e}=\frac{\sigma_{1}^{e}}{\left.{\left(\sigma \right.}_{\max}^{0}\right)}$$

Here, $\sigma_{\max}^{0}$ represents the MPS, while $\sigma_{1}^{e}$ corresponds to the MPS as obtained from our finite element model. It is important to note that cell cracks occur when the value of f surpasses unity (f > 1).

### Calculation and analysis

The initial crack load for dentin and restoration was measured for each model. A Pearson correlation test was conducted using SPSS 23.0 (SPSS Inc., Chicago, IL) to analyze the relationship between dentin initial crack load and restoration initial crack load. Statistical significance was defined as a *p*-value of <0.05.

The distribution of MPS, strain, and displacement within dentin, crack lines, restorations, pulp, and enamel was visualized. Unstressed areas were depicted in dark blue, while more stressed areas were represented in varying shades of red.

## Result

### Initial crack load, MPS, strain, and displacement of dentin

**FIGURE 4:**
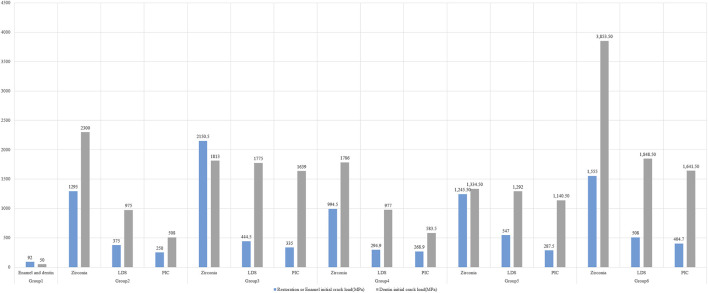
Initial Crack loads on restorations and dentin.

The results of the study are presented in Figure 4, which shows the dentin crack initial loads in the control group without any prosthetic protection, the crack was observed to continue to develop at a load of only 50 N. However, the use of restorations covering the occlusal surface greatly improved the resistance to continued crack development. It was also observed that the higher the strength of the material used, the better the ability to protect the hidden cracked tooth from further cracking.

**FIGURE 5:**
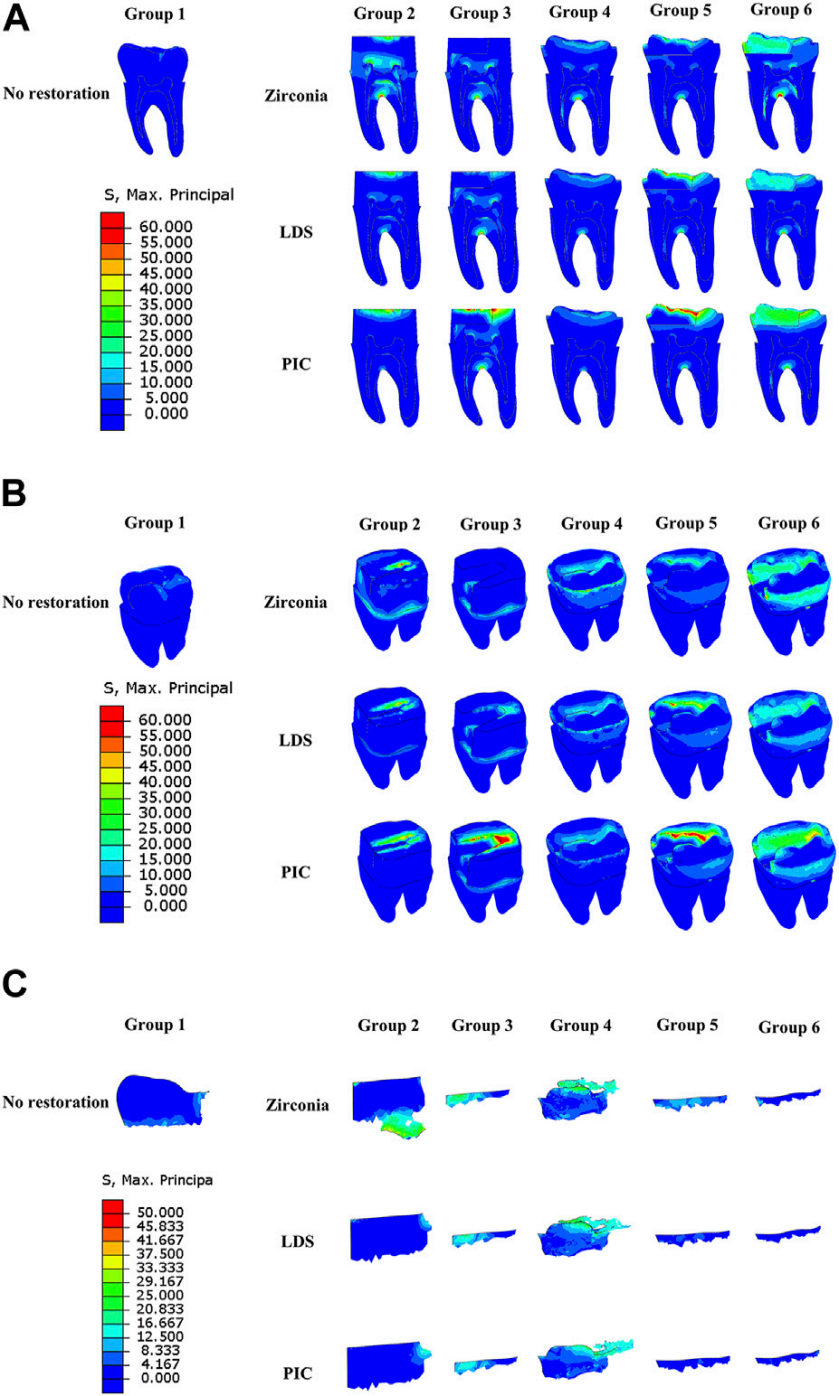
Distribution of maximum principal stresses in six different groups of model dentin during crack propagation. **(A)** Longitudinal section of dentin. **(B)** Dentin surface. **(C)** Crack line. The red area indicates regions of elevated stress, while the dark blue area represents regions of reduced stress.

Figure 5 illustrates the concentration of MPS on the dentin, primarily at the interface between the restoration and the dentin, as well as at the root bifurcation. The distribution of MPS showed that the area at the top of the crack development exhibited the highest MPS values on the crack line. Additionally, the dental tissue at the root bifurcation demonstrated the highest MPS values, although no fissure production was observed.

**FIGURE 6:**
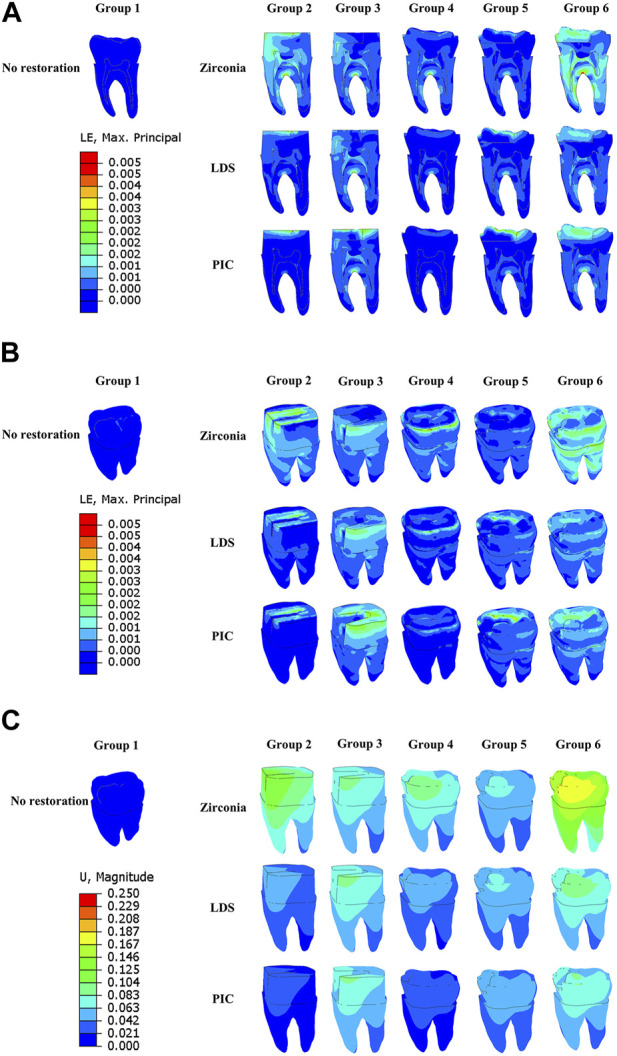
Strain and displacement distributions of six distinct groups of model dentin during crack propagation. **(A)** Strain distribution on the longitudinal section of dentin. **(B)** Strain distribution on the dentin surface. **(C)** Displacement distribution of dentin. The red area denotes regions of heightened strain or displacement, while the dark blue area signifies regions of diminished strain or displacement.

In Figure 6, the variation pattern of dentin strain was not clearly discernible and did not show a significant correlation with MPS. However, Figure 6 also revealed that dentin covered by oxidized restorations exhibited the highest displacement magnitude within the same group, while dentin covered by PIC restorative restorations exhibited the lowest displacement. Notably, there was a significant difference in dentin displacement between Groups 3 and 6 compared to the other groups.

### Initial crack load and MPS, strain, and displacement of restorations

Figure 4 showcases the initial crack load and the mechanical properties of different restorative materials. Among the three materials studied, zirconia exhibits the highest modulus of elasticity and tensile stress, indicating its superior resistance to fracture. In terms of restoration design, Group 3 restorations demonstrate the highest resistance to fracture, while Group 4 restorations display the lowest resistance.

**FIGURE 7:**
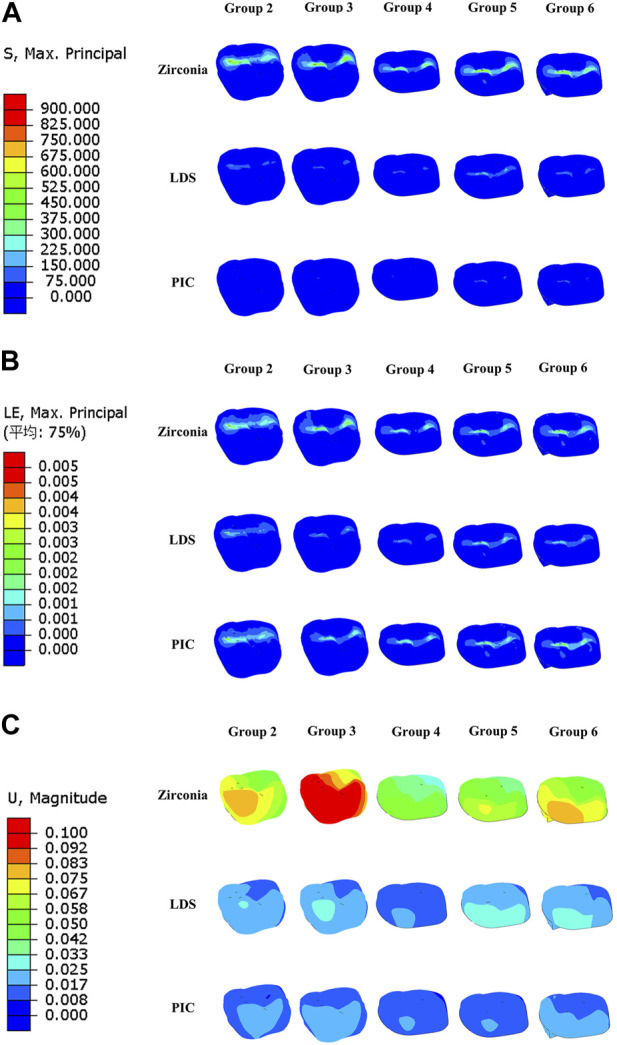
Distribution of maximum principal stress, strain, and displacement in six different groups of model restorations during crack propagation. **(A)** Maximum principal stress distribution in the restorations. **(B)** Strain distribution of restorations. **(C)** Displacement distribution of restorations. The red area denotes regions of high strain or displacement, while the dark blue area signifies regions of low strain or displacement.

Turning to Figure 7 highlights the unique patterns observed in the distribution of MPS, strain, and displacement among various types of restorations. Specifically, the crack of the zirconia restoration exhibits the highest concentration of MPS, suggesting enhanced resistance to crack propagation. In contrast, the crack of the PIC restoration displays the lowest MPS concentration. Interestingly, the strain cloud surrounding the cracks of both zirconia and PIC restorations exhibits relatively high strains, indicating the potential for localized deformation. Conversely, the cracks of LDS restorations exhibit low strains. Examining displacement, zirconia restorations demonstrate the highest displacement, suggesting a greater degree of movement or deformation, while the PIC restorations exhibit the lowest displacement.

### MPS, strain, and displacement of pulp

**FIGURE 8:**
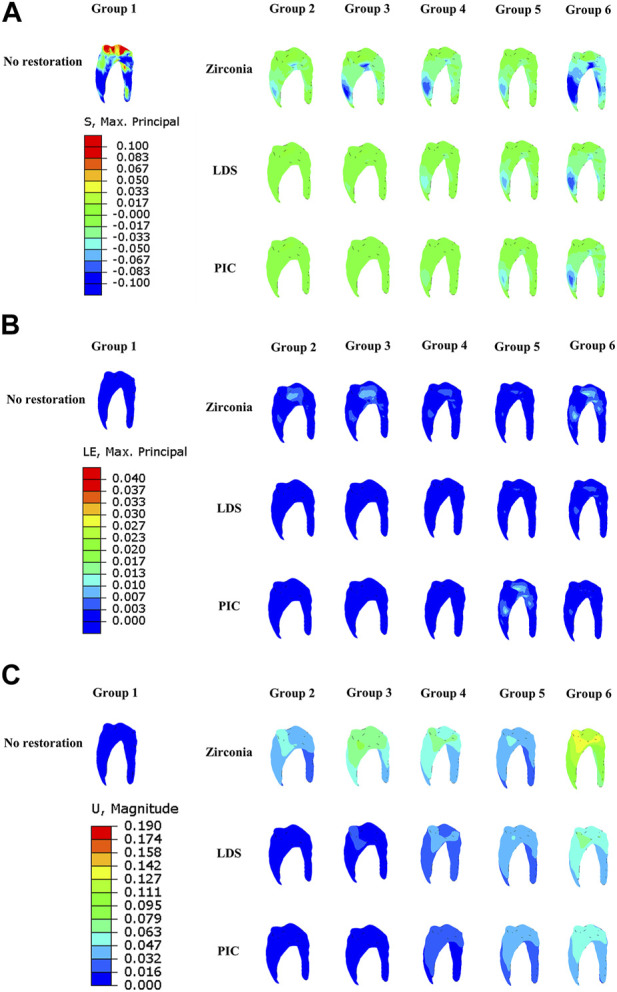
Distribution of maximum principal stress, strain, and displacement in six distinct group of models pulp during crack propagation. **(A)** Maximum principal stress distribution in the pulp. **(B)** Strain distribution of dental pulp. **(C)** Displacement distribution of dental pulp. The red area indicates regions of high strain or displacement, while the dark blue area represents regions of low strain or displacement.

Figure 8 illustrates notable observations regarding MPS concentration, strain, and displacement in relation to the pulpal tissue. High MPS concentration was observed primarily at the top of the control pulp, while a partial low MPS concentration was noted in the root region of the pulp in models with zirconia restorations and in Group 6. Similarly, strain analysis in Figure 8 revealed a concentration of strain in the pulp across most models with zirconia restorations, as well as in select other models, such as Group 5 (PIC). Furthermore, Figure 8 demonstrated that the displacement of the pulp was most prominent in the model with zirconia restorations within the same group, with a more pronounced tendency for pulp displacement observed in Group 6.

### MPS, strain, and displacement of enamel

**FIGURE 9:**
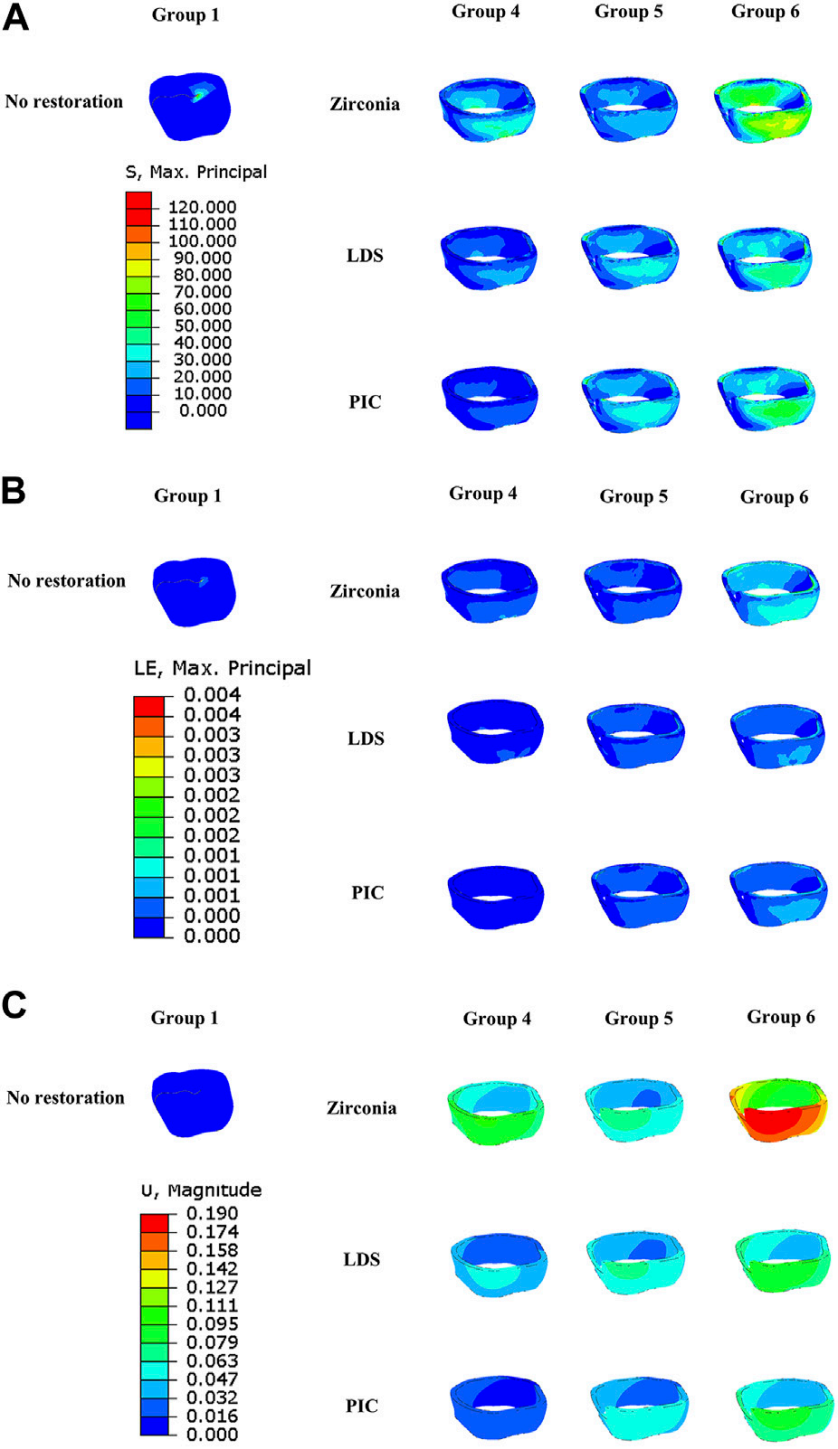
Distribution of maximum principal stress, strain, and displacement in six distinct groups of model enamel during crack propagation. **(A)** Maximum principal stress distribution in the enamel. **(B)** Strain distribution of enamel. **(C)** Displacement distribution of enamel. The red area indicates regions of high strain or displacement, while the dark blue area represents regions of low strain or displacement.

In Figure 9, the distribution of MPS, strain, and displacement in the enamel of groups 1, 4, 5, and 6 is presented. In the control group, the MPS concentration is observed around the jaw fissure of the enamel. Interestingly, larger MPS values and displacements are observed in both the zirconia restoration models and group 6. Additionally, the strain cloud reveals a higher strain in group 6.

### Insurance role of the restoration

As depicted in Figure 4, the initial load for restoration or enamel was lower than the initial load for crack propagation in dentin in the control group and Group 3. However, in the remaining experimental groups, the crack propagation initial loads in dentin surpassed those in the restorations. This indicates that the restoration plays a crucial role in protecting the tooth. The correlation analysis in Table 3 showed a significant positive correlation (*r* = 0.617, *p* < 0.05) between restoration initial load and dentin initial load.

**TABLE 3:** Pearson correlation analysis between restoration initial crack load and dentin initial crack load.

	—	Restoration initial crack load	Dentin initial crack load
Restoration initial crack load	*r*	1	0.617*
	*p*	—	0.014
Dentin initial crack load	*r*	0.617*	1
	*p*	0.014	—

*p< 0.05.

### Crack pattern

As shown in Figure 5, the morphology of dentin crack development has a similar extension pattern within the same restoration type but varies between restoration protocols. The initial crack area and morphology of groups 3, 5, and 6 were exactly the same and smaller than the other groups. We observed that the cracks in these groups developed along the bottom of the original cracks toward the buccal side under stress. In the control group, where there was no restorative protection, the original fissure of the dentin started to expand in the distal mid-position of the jaw surface. In Group 2, the initial location of crack propagation and extension pattern were similar for LDS and PIC, both of which started to develop in the middle of the far-middle of the crack and extend towards the far-middle. However, the fissure extension of zirconia material in Group 2 was mainly concentrated at the bottom of the fissure near the pulp chamber, with a small amount of fissure extension expected to start in its far middle but was not obvious. The crack propagation of Group 4 extended from the dentin on the crown side and extended over the surface of the dentin, forming a cloud-like morphology.

## Discussion

Computational approaches have proven advantageous in fracture studies of brittle materials with complex geometries, such as cracked tooth models. Experimental settings are unable to accurately model tooth cracks with a thickness of only 17 μm, and problems arising from specimen asymmetry and misalignment make fracture behavior uncontrollable, resulting in dispersed test results (Zhang et al., 2016). However, this disadvantage can be eliminated by using the XFEM independent element crack surface and crack front morphology to simulate crack evolution without the need to re-mesh during crack propagation (Belytschko et al., 2009).

Recent studies (Zhang et al., 2019; Kim et al., 2021; Lin et al., 2022) have primarily focused on the stress distribution of teeth under load and the properties of restorative materials, without considering the possibility of restorative material fracture and how stress might change after fracture. In our study, we create a model of a cracked first mandibular molar and applied a force load to it in order to study the effect of the combination of restorative materials and restorative methods on the evolution and extension of crack. We used the XFEM to simulate the cracking process of different restorative materials and restorative methods of dentin simultaneously under extreme loads, a novel approach not previously utilized in this context. Both restorative materials and dentin were treated as quasi-brittle materials susceptible to tensile fracture, with compressive resistance far exceeding their tensile strength. Intrinsic damage occurred when loaded beyond maximum tensile strength, preceding macroscopic fracture. Overall, our study provides insights into the complex mechanisms underlying the cracking process of restorative materials and dentin, and may inform future developments in the field of restorative dentistry.

Cracked tooth is a prevalent oral disease, and biting pain is a common clinical symptom associated with it (Signore et al., 2007; Banerji et al., 2017). The mechanics of cracks play a crucial role in understanding this relationship. When an individual applies pressure or bites down on the tooth, the crack may open slightly, causing movement and separation of the fractured segments. This movement can irritate the pulp tissue within the tooth, resulting in pain (Hilton et al., 2018; Longridge and Youngson, 2019). In our study, we intentionally placed a crack 2 mm away from the pulp chamber, as shown in Figures 3, 4. We observed that in teeth with occult fractures, crack propagation occurred at a low load of 50 N without any restoration. Furthermore, in Figure 8, we observed a larger distribution of MPS at the top of the pulp in Group 1 when subjected to a 50 N load, unlike other model groups. This indicates that if a cracked tooth is left unprotected, the pulpal region is susceptible to occlusal pain due to stress, even with a smaller load. However, with the protection provided by a restoration covering the occlusal surface, the initial load on dentin could be increased to a minimum of 508 N. It is important to note that previous studies (Ye et al., 2015) have reported occlusal forces ranging from 335 to 1,727 N in the maximum occlusal position, with average occlusal forces for molars ranging from 107 to 156 N. Therefore, it is evident that when the crack develops closer to the pulp chamber (within or less than 2 mm), it can easily extend under normal occlusal loading experienced by molars (107–156 N). These findings, similar to previous studies (Kim et al., 2013; Wu et al., 2019), highlight the importance of prompt diagnosis and treatment of cracked teeth to prevent further damage to the tooth structure. Moreover, the use of a protective restoration significantly enhances the tooth’s resistance to crack initiation and extension, providing an effective approach to managing this common dental issue (Chen et al., 2021; de Toubes et al., 2022).

In crack propagation analysis, the MPS is a very important parameter that can be used to determine if there is a possibility of crack extension in the material and the direction and rate of crack extension (Wang et al., 2016). The MPS is the one that is the largest among the stresses at a point, along any direction. In a cracked material, the MPS will usually occur near the crack tip (Yamaguchi et al., 2018). Figure 5 in our study illustrates that in dentin, the MPS is primarily concentrated at the root bifurcation and the junction between the restoration and dentin. Notably, no cracking was observed at the root bifurcation in any of the models. However, an extension of the crack line was observed at the restoration-dentin junction in groups 1, 2, and 4. Additionally, Figure 5 demonstrates that the MPS reaches its peak precisely at the tip of the crack line development. This phenomenon can be attributed to the stress concentration that occurs at the crack tip due to non-uniform stress distribution resulting from external loading. This non-uniform stress field significantly influences the expansion of cracks. In contrast, the tooth structure at the root bifurcation remains relatively intact and demonstrates greater resistance to cracking compared to the tooth tissue surrounding the crack line, despite experiencing higher MPS. This observation suggests that the root bifurcation’s structural integrity plays a crucial role in impeding crack formation, despite being subjected to elevated stress levels.

Figures 5, 6 clearly demonstrate a positive correlation, to a certain extent, between the displacement of dentin and the applied load, displaying an observable linear trend. Nonetheless, it is important to note that no significant linear relationship was observed between MPS and strain in the dentin. This lack of correlation can potentially be attributed to the presence of pre-existing cracks within the dentin prior to experimentation, as well as the development of additional cracks during the loading process. These findings align with those of previous researchers (Emamian et al., 2021), who have also described dentin as a material exhibiting near-linear behavior within a limited strain range. However, under conditions of higher stress and strain, dentin exhibits nonlinear behavior.

Previous research (Robati Anaraki et al., 2019; Peskersoy and Sahan, 2022) has primarily focused on assessing the stress distribution on teeth or materials under load, considering various physical characteristics such as elastic modulus and Poisson’s ratio of restorative materials, while neglecting the fracture resistance of the materials. To address this limitation, we incorporated fracture-related parameters into our analysis of the restorative materials. Moreover, using XFEM, we simulated the initiation and propagation of fractures in the restoration during the loading process. Our findings, as depicted in Figures 4, 7, consistently align with previous studies (Shahmoradi et al., 2020), demonstrating that zirconia restorative materials with high elastic modulus, tensile strength, and fracture toughness exhibit the highest crack load (ranging from 1,295 to 2,150.5 N) within the same model. To further investigate the protective effects of the materials on dental tissues, we incorporated fracture toughness parameters for both the restorative and dental materials within the XFEM process. This allowed us to simulate the protective role of the restorative material after fracture. Figure 4 confirms our study’s alignment with prior experimental findings (Dejak et al., 2012; Alsadon et al., 2017), illustrating that dental materials with a high elastic modulus, such as zirconia, offer superior protection against tooth fracture compared to those with a low elastic modulus, like PIC. Moreover, our analysis, supported by Pearson correlation analysis (Table 3), reveals a significant positive correlation between the initial load on the restoration and dentin. This correlation emphasizes the favorable relationship between the restoration’s strength and its ability to protect dental tissues. Additionally, we conducted a comprehensive examination of the strain and displacement of the restorative material. Figure 7 illustrates that the displacement trend of the restoration mirrors that of the dentin shown in Figure 6, with more pronounced displacement observed under higher loads. Notably, the strain distribution contour maps indicate that LDS (a specific restorative material) exhibits lower strain at the site of cracking compared to zirconia and PIC. In conclusion, our findings offer valuable insights into the impact of material properties on the resistance to crack propagation and underscore the importance of considering the fracture behavior of both the restorative material and dentin when evaluating the efficacy of dental materials in treating cracked teeth.

In our investigation, the design of dental restorations was found to influence their ability to withstand continuous crack propagation in dentin. We also discovered a correlation between the size of the crack area and the dentin’s fracture resistance. As shown in Figure 4 the dentin in Group 6 exhibited the highest fracture initial load, while the dentin in Group 2 demonstrated the lowest fracture initial load. When comparing different prosthetic designs, we observed that the group with a larger crack area (Group 2, Group 4) had a smaller crack initial load compared to the group with a smaller crack area (Group 3, 5, 6). On the same type of restoration (Group 2, 3, 4, 5), the resistance to cracking is greater in the groups where the cracks are mostly eliminated and replaced by filler (Group 3, 5), as compared to the groups without crack elimination (Group 2, 4). The issue of whether to eliminate crack lines or not remains a subject of ongoing debate in dental research. Some experts advocate for complete elimination of cracks (Abbott and Leow, 2009; Batalha-Silva et al., 2014), while others argue for partial elimination (Ito et al., 1998). Conversely, a group of authors proposes maximizing the preservation of tooth structure and suggests cuspal coverage restorations as a means to protect the teeth without completely eliminating the cracks (Griffin, 2006; Soares de Toubes et al., 2020). Previous studies have reported higher rates of pulp vitality preservation (93%) when cracks were eliminated or reduced in size (Krell and Rivera, 2007; Yamaguchi et al., 2018). However, crown restorations without eliminating crack lines resulted in lower rates of pulp vitality preservation, ranging from 79% to 71% (Kanamaru et al., 2017; Wu et al., 2019). The variation in the preservation rate of vital teeth mentioned above could potentially stem from differences in the inclusion criteria employed across various studies. However, from an infection resistance perspective, even with both sides of the crack line sealed, significant inflammation or residual toxins may still be present, as bacterial toxins can penetrate deep cracks near the pulp chamber, potentially leading to complications such as irreversible pulpitis or pulp necrosis (Berman and Kuttler, 2010; Ricucci et al., 2015). Furthermore, the application of resin infiltration to eliminate crack lines in a model of caries-associated hidden cracks in maxillary first molars resulted in the restoration of crack initiation load and fracture load to 75% and 90% of the values observed in corresponding intact tooth models, respectively (Shahmoradi et al., 2022). Based on these clinical studies and our findings, we hypothesize that the presence of hidden cracks compromises the structural integrity of the tooth, with a larger crack area corresponding to a lower proportion of intact and uniform sections within the tooth. This conclusion aligns with the findings of other researchers (Kim et al., 2021; Shahmoradi et al., 2022).

Recently, it had been reported that crack propagation was expected to follow a direction parallel to the cusp incline (Homewood, 1998) with stresses concentrated at the restoration-tooth interface following loading (Roh and Lee, 2006). However, our study has revealed different trends in crack extension. In particular, Groups 3, 5, and 6 exhibited shorter crack lines, with a similar direction of fissure extension despite differences in the restorations used and the materials employed. In contrast, the crack extension pattern observed in Group 4 may be attributed to the jaw veneer restoration used, which fully covered the cusps and did not extend significantly in the axial position. Consequently, a cloudy crack developed, with most of the stress concentrated on the restoration-tooth interface. Regarding the LDS and PIC used in Group 2, similar crack extension patterns were observed, but zirconia differed in this respect. This disparity may be due to zirconia’s high fracture resistance, which allows it to absorb horizontal force, with the load mainly transferred vertically upwards to the tooth. In contrast, LDS and PIC have inherent limited strength, and after the crown fractures, horizontal forces are transferred to the tooth, causing fractures in the dentin to expand horizontally.

Currently, our study has some limitations. Firstly, dentin was treated as a linearly elastic, isotropic material, which does not fully capture its anisotropic, viscoelastic mechanical behavior. To improve the accuracy of our simulation results, a fracture mechanics approach that takes into account the microstructure of dentin should be employed. Further experiments are necessary to refine our understanding of dentin’s mechanical properties. Secondly, the complexity of our research model, which involves multiple materials and requires the definition of cracking, led to difficulties in achieving convergence during the analysis. To overcome this challenge, we employed the principle of engineering equivalence and applied force to the occlusal surface at a 45-degree angle. While this approach was simple and effective, it is not representative of realistic occlusal contact. A more accurate simulation would involve establishing a realistic occlusal contact (Duanmu et al., 2021) or using a simplified rigid ball to form an occlusal contact, which would allow us to output the cracking load of the corresponding contact material (Wan et al., 2019). Thirdly, an initial three-dimensional crack was set up at the beginning of the experiment. However, due to limitations in Abaqus software, the initial crack had to be reduced to a two-dimensional plane when specifying a three-dimensional solid. As a result, the prefabricated crack is equivalent to the macrostructure of the tooth tissue and behaves similarly to the neighboring tissue in terms of stress levels. Further research should explore the influence of the initial crack location and orientation on the prognosis of a full-crown restoration.

## Conclusion

A deep fissure in a cracked tooth can cause the fissure to expand under a small load, which can lead to further damage. Restorative interventions that cover the entire cusp have been shown to be effective in enhancing the fracture resistance of cryptically fractured teeth. The fracture resistance of the restorative material is a critical factor in protecting the tooth from further cracking, with stronger materials offering better protection. The elimination of crack lines has been shown to improve the fracture resistance of cracked teeth, further enhancing the efficacy of restorative interventions.

## Data Availability

The raw data supporting the conclusion of this article will be made available by the authors, without undue reservation.
